# What is the Role of the Horizontal Transmission of Hepatitis B Virus Infection in Young Adult and Middle-Aged Roma Population Living in the Settlements in East Slovakia?

**DOI:** 10.3390/ijerph17093293

**Published:** 2020-05-09

**Authors:** Sylvia Drazilova, Pavol Kristian, Martin Janicko, Monika Halanova, Dominik Safcak, Patricia Denisa Dorcakova, Maria Marekova, Daniel Pella, Andrea Madarasova-Geckova, Peter Jarcuska, HepaMeta Team

**Affiliations:** 1 Department of Internal Medicine, Hospital Poprad and Faculty of Medicine, P.J. Safarik University, 058 01 Poprad, Slovakia; drazilova.s@nemocnicapp.sk; 2 Department of Infectology and Travel Medicine, Faculty of Medicine, P.J. Safarik University and L. Pasteur University Hospital, 040 01 Kosice, Slovakia; patricia.denisa.dorcakova@student.upjs.sk; 3 2nd Department of Internal Medicine, Faculty of Medicine, P.J. Safarik University and L. Pasteur University Hospital, 040 11 Kosice, Slovakia; martin.janicko1@upjs.sk (M.J.); peter.jarcuska@upjs.sk (P.J.); 4 Department of Epidemiology, Faculty of Medicine, P.J. Safarik University, 040 01 Kosice, Slovakia; monika.halanova@upjs.sk; 5 East Slovakia Oncology Institute and Faculty of Medicine, P.J. Safarik University, 040 01 Kosice, Slovakia; saffkov@gmail.com; 6 Department of Medical and Clinical Biochemistry, Faculty of Medicine, P.J. Safarik University, 040 11 Kosice, Slovakia; maria.marekova@upjs.sk; 7 2nd Department of Cardiology, East Slovak Institute of Cardiovascular Diseases and Faculty of Medicine, P.J. Safarik University, 040 11 Kosice, Slovakia; daniel.pella@upjs.sk; 8 Department of Health Psychology, Faculty of Medicine, P.J. Safarik University, 040 11 Kosice, Slovakia; andrea.geckova@upjs.sk

**Keywords:** Roma population, hepatitis B infection, horizontal transmission, hepatitis B prevalence

## Abstract

*Background*: The aim of our work is to objectify the manner of transmission of HBV infection in young adult and middle-aged Roma people who live in the settlements. *Methods*: We used data from the cross-sectional study HepaMeta. We analyzed Roma people living in the settlements in East Slovakia, who have had HBsAg and anti HBc IgG antibodies examined. *Results*: We analyzed a cohort of 452 Roma participants with a mean of age 34.67 ± 9.14 years—159 (35.2%) were males. HBsAg positivity was diagnosed in 12.4% and the presence of anti HBc IgG antibodies was confirmed in 52% of participants. Prevalence of HBsAg positivity increases significantly with higher age, (*p* = 0.026), as well as the presence of anti HBc IgG antibodies (*p* < 0.0001). The prevalence of HBsAg positivity has doubled and anti HBc IgG positivity has tripled within two decades (<25 years vs. 35–45 years) in Roma settlements in East Slovakia. *Conclusions*: These findings allow us to express an opinion that horizontal transmission in adulthood may play an important role in the spreading of HBV infection.

## 1. Introduction

Hepatitis B virus (HBV) predominantly impairs liver parenchyma, in which acute or chronic inflammation occurs. Acute hepatitis B infection may spontaneously resolve or become chronic. Chronically infected patients with HBV may be inactive carriers or may develop chronic hepatitis B. Chronic hepatitis B may progress to liver cirrhosis and as a consequence of liver cirrhosis, some patients develop hepatocellular carcinoma (HCC). HCC may also occur without liver cirrhosis in patients with long-term HBV infection [1].

Although HBsAg is present in a wide variety of body secretions, only blood, semen and saliva have been shown to be infectious. Hepatitis B is transmitted by two basic ways of transmission:

1. Vertical: mother to child—perinatal
2. Horizontal: by blood or sexual intercourse [2]

There are three routes of transmission of infection from an infected mother to a child: transplacental transmission in the uterus, transmission during delivery and postpartum transmission during childcare and breastfeeding. Perinatal transmission is responsible for most of the transmission of HBV infection worldwide [3].

The risk of transmission of HBV infection through sexual intercourse increases with regular sexual contact with an HBV-infected person, frequent partner swapping and homosexual intercourse. Homosexual intercourse between men is associated with a high risk for transmission of infection [4].

Hepatitis B virus can be transmitted via the bloodstream as follows: transfusion of blood and blood derivatives, needle stick or blood splatter from a person infected with hepatitis B virus or an undiagnosed person, infected blood transfusion products, open injuries and scratches, bloody surgical and microsurgical procedures, intravenous drug use, tattoo and piercing, hemodialysis, manicure and pedicure, and sharing of razors and toothbrushes [2,5,6].

The age at the time of HBV infection, HBeAg/anti HBe status, and immunological mechanisms are responsible for the transition of hepatitis B virus infection to chronicity. The group who is most at risk are neonates born to HBeAg-positive mothers; the perinatal transmission of HBV in these children is 70%–90% and 90% of them remain chronically infected. Whereas, in neonates born to HBeAg-negative mothers, the perinatal transmission of HBV infection is 10%–40% and 40%–70% of infected children develop chronic infection [2,7]. Children who were born to HBsAg-positive mothers, who have not been infected perinatally, have a high risk of infection in childhood as well. One study showed that up to 40% of such children born to HBeAg negative mothers are infected within the first 5 years of life [8]. With higher age, the proportion of patients progressing to chronic HBV infection decreases; 20%–60% of children infected between the age 1–5 years and 5%–10% of older children and adults progress to chronicity [3].

There are approximately 292,000,000 people with chronic HBV infection, which represents approximately 3.9% of the world’s population [9]. About 887,000 people die every year due to chronic hepatitis B complications—mainly decompensated liver cirrhosis and HCC [10]. Countries can be divided into three groups according to the hepatitis B prevalence:

1. Countries with low prevalence of hepatitis B (up to 2%)
2. Countries with moderate prevalence of hepatitis B (2%–8%)
3. Countries with a high prevalence of hepatitis B (over 8%)

About half of the total population lives in areas with a high prevalence of hepatitis B. In countries with a high prevalence of hepatitis B, 70%–90% of the population have serological evidence of previous contact with hepatitis B [11]. In almost all cases, the infection occurred perinatally or during early childhood, resulting in a high incidence of chronic hepatitis B in this population. Chronic hepatitis B has a strong association with an HCC; therefore, these areas also have a high mortality due to HCC. Hepatitis B was highly endemic in developing countries with a high population number in Southeast Asia and Sub-Saharan Africa. Thanks to an effective vaccination program, the prevalence of chronic HBV infection has been reduced in many of the concerned countries [12,13]. In countries with moderate prevalence of hepatitis B, 10%–60% of the population have serological evidence of hepatitis B contact, combining perinatal transmission, HBV infection in early childhood, and horizontal transmission in adulthood [14]. These are the countries of Eastern and Southern Europe, the Middle East, Japan and a part of South America. The most developed countries in the world (USA, New Zealand, Australia, Western Europe) have a low prevalence (less than 1%) and infection occurs in adulthood, especially in the high-risk population: intravenous drug users, homosexuals, heterosexuals with risky behavior, healthcare workers, hemodialysed patients and blood transfusion recipients. An estimated 5%–7% of the population has been exposed to the virus sometime during life [11]. The worldwide prevalence of hepatitis B is shown in Table 1, adjusted according to [2,15].

The aim of our work is to objectify the route of transmission of HBV infection in young adult and middle-aged Roma people who live in the settlements.

## 2. Methods

We used data from the cross-sectional study HepaMeta, which was carried out in 2011, while studying Roma people living in the segregated settlements in East Slovakia.

The recipients filled out questionnaires containing data about lifestyle and risky behavior that can lead to HBV infection, and we performed anthropometric examinations and blood and urine laboratory sampling on them.

All of them were screened for HBsAg and anti HBc IgG in plasma, testing was performed by Enzygnost (Siemens, Eschborn, Germany). In case of HBsAg positivity, the participants were considered to be chronically infected with HBV. Anti HBc IgG positivity is present in chronic HBV infection or after overcoming hepatitis B (clinically manifested or inapparent) without transition to chronicity, so the positivity of anti HBc IgG antibodies was considered to be a serological marker of contact with HBV.

A detailed description of the study design and the methods used was published by Madarasova-Geckova et al. (2014) [16].

The study was approved by the Ethics Committee of P.J. Safarik University, Faculty of Medicine in Kosice, Slovakia. Participation in the study was fully voluntary and anonymous. Detailed information about the study and its procedures was given to all respondents, and informed consent was obtained prior to the medical examination.

### Statistical Analysis

Interval variables are presented as mean ± standard deviation or median ± interquartile range where appropriate. A simple comparison of interval variables distribution between two groups was performed by a T-test and by multiple-groups by ANOVA. A comparison of categorical variables between multiple categories was performed by a chi-squared test with Yates continuity correction or Fisher exact test where appropriate. Trends and Odds ratios were analyzed by binary logistic regression with anti HBc IgG and HBsAg as dependent variables and age categories as categorical independent variables. Multivariate regression was adjusted for age and sex.

## 3. Results

### Description of Study Population

The study population comprised of 452 Roma participants with a mean of age 34.67 ± 9.14 years—159 (35.2%) were males. Ten participants were missing data on HBsAg and eleven on anti HBc IgG antibodies.

Table 2 summarizes the risk factors for HBV infection transmission in the entire patient cohort. Table 3. compares the risk factors for HBV infection transmission between each group. Younger Roma people were more likely to have sex with more than 4 sexual partners (*p* = 0.04) throughout their lives, while older Roma people had a greater number of total tattoos (*p* = 0.0003) and tattoos that were done privately (*p* = 0.0001).

Hepatitis B serological markers and age.

**Figure 1:**
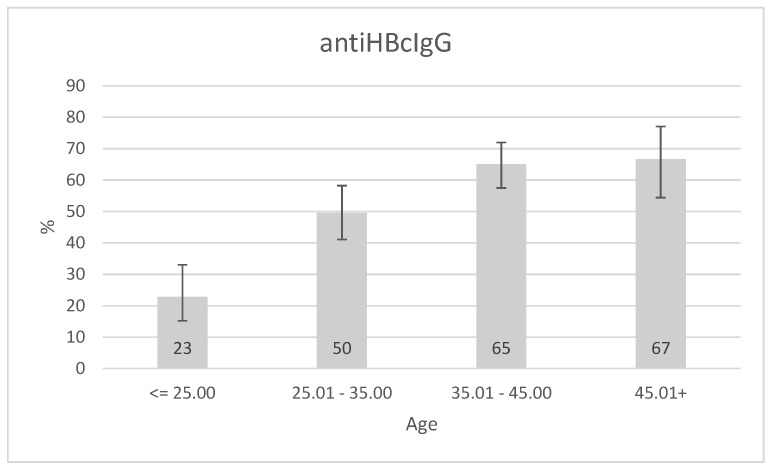
Anti HBc IgG positivity prevalence according to age, error bars represent 95% CI. *p* for trend <0.0001.

Roma participants positive for anti HBc IgG were significantly older (37.14 ± 8.01 vs. 31.85 ± 9.5 years) than anti-HBc IgG-negative Roma participants, *p* < 0.0001. Therefore, we looked closer at the relationship between age and Hepatitis B serological markers. When the study participants were organized into 10-year brackets, a clear increase of anti HBc IgG prevalence was observed in the unselected cohort (*p* < 0.0001) (see Figure 1).

**Figure 2:**
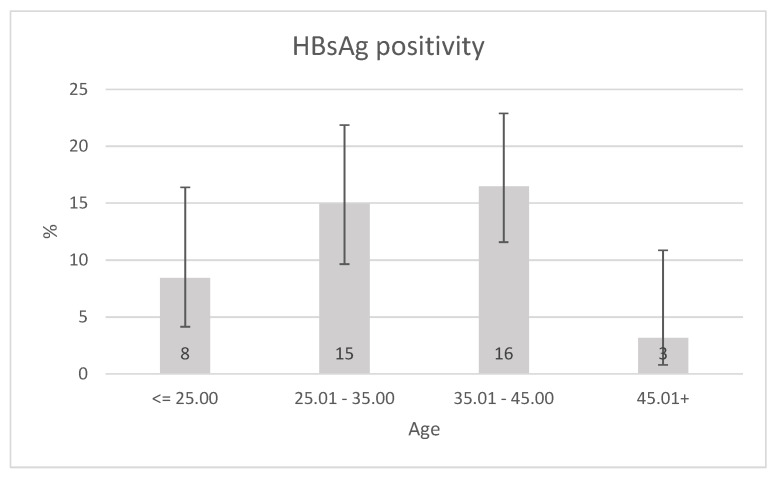
HBsAg positivity prevalence according to age, error bars represent 95% CI, *p* for trend 0.047.

Despite there was no difference in age between HBsAg-positive and -negative groups, we extended this analysis to HBsAg as well (Figure 2). We observed a statistically significant difference in HBsAg positivity across age categories (*p* = 0.026), with an obvious pattern of HBsAg positivity increase with a maximum of HBsAg prevalence between the 35th and 45th year of life and decrease in the 45+ year group (*p* for trend 0.047), which may be related to the route of transmission.

A comparison of imprisonment, drug use, blood transfusion, sexual behavior and tattoo procedure between HBsAg-positive and -negative Roma people is shown in Table 4.

A comparison of imprisonment, drug use, blood transfusion, sexual behavior and tattoo procedure between anti-HBc IgG-positive and HBc IgG-negative Roma people is shown in Table 5.

Association of the variables that were significantly different between HBsAg-positive vs. HBsAg-negative and anti-HBc IgG-positive vs. anti-HBc IgG-negative groups was also analyzed in multivariate regression adjusted for age and sex.

Significant differences between HBsAg-positive and -negative groups were observed only in males. In multivariate regression, males were associated with HBsAg positivity with OR 2.083 (95% CI 1.176–3.690); *p* = 0.012 adjusted for age (see Table 4).

Significant differences between anti-HBc IgG-positive and anti-HBc IgG-negative groups were observed in age, imprisonment and tattoo presence. In multivariate regression adjusted for sex, higher age was significantly associated with anti HBc IgG positivity (OR for 1-year increment was 1.080; 95%CI 1.054–1.107; *p* < 0.0001). However, no significant association was observed between imprisonment or tattoo presence after adjustment for age and sex (see Table 5).

## 4. Discussion

We evaluate epidemiological aspects of HBV transmission in the adult Roma population living in the segregated settlements, in our work. HBsAg positivity (indicating chronic HBV infection) was diagnosed in 12.4% of patients and anti HBc IgG antibodies (indicating long-term presence of HBV in the body or overcome hepatitis B in the past) were found in 52.8% of patients. The prevalence of chronic HBV infection in Roma people is significantly higher than in the adult majority population of East Slovakia (2.8%) [6]. The prevalence of chronic hepatitis B among Roma people living in the settlements ranks this community among areas with a high prevalence of chronic HBV infection, see Table 1. A similar prevalence of chronic HBV infection is found in migrant communities from Southeast Asia in the United States [17].

Some HBsAg-positive patients may lose HBsAg during the long-term follow-up. In Western countries where HBV infection is predominantly acquired in adulthood, the annual HBsAg clearance is 1%–2%. However, in countries with a high prevalence of chronic HBV infection, which is mostly acquired perinatally or in younger age, the loss of HBsAg is 0.05%–0.8% annually. Patients who lose HBsAg, or even produce anti HBs antibodies, have a better prognosis than HBsAg-positive patients, but some may develop liver cirrhosis decompensation or HCC [1]. A very interesting finding is the relatively low prevalence of chronic HBV infection in Roma people over 45 years old, although the positivity of anti HBc antibodies in this age group continued to increase. We cannot clearly explain this finding. We can only speculate that some of them have lost HBsAg and some have died due to complications of chronic hepatitis B at a younger age; however, we cannot objectify this assumption due to lack of data. The finding of a low prevalence of HBsAg positivity in this age group is also certainly influenced by a small number of patients assessed at this age range.

In our work, we also analyze the risk factors of transmission of HBV infection in adulthood. HBV infection may be transmitted by blood or sexual intercourse in this age group. Less than 10% of Roma people in our cohort use condoms always or almost always during sexual intercourse, with no difference in the use of this barrier contraception method between age groups. The use of condom prevents leakage of HBV [18]. Regular use of condom in a Peruvian study reduced the incidence of anti HBc positivity by almost three times [19]. HBV infection is more common in partner rotation, younger Roma people reported more promiscuous behavior than older. Tattoos, prison stay, intravenous drug use and blood transfusion were observed as risk factors for parenteral transmission of HBV infection [20,21]. Tattoos increase the risk of transmission of hepatitis B by about half and in patients with risky behavior by more than 60%, while tattoos done privately, which are often performed with unproperly sterilized tattoo instruments, are more dangerous [21]. In our cohort, we observed highly risky behavior for HBV transmission by blood in Roma people: nearly 40% of Roma people had a tattoo, where almost all of them had a tattoo done privately. The number of Roma people with a tattoo increases with higher age. Over 10% of Roma people have been imprisoned and almost 17% have had a blood transfusion. Roma people living in settlements in East Slovakia rarely use intravenous drugs. Significant differences between anti-HBc IgG-positive and anti-HBc IgG-negative groups were observed in age, imprisonment and tattoo presence; however, no significant association was observed between imprisonment or tattoo presence in multivariate analysis after adjustment for age and sex.

In areas with a high prevalence of chronic HBV infection (more than 8% prevalence of HBsAg), a high prevalence of anti HBc antibodies is a typical finding as well. The vast majority of patients are infected at perinatal or preschool age, while in some areas perinatal transmission predominates and in other areas the parenteral transmission in preschool age [2,6,22]. Despite the high prevalence of chronic HBV infection, the Roma people living in the settlements differ from other areas with a similar prevalence of HBsAg positivity. With the increasing age, the prevalence of HBsAg positivity, (*p* = 0.026) as well as the presence of anti HBc IgG antibodies, is increasing significantly (*p* < 0.0001). The prevalence of chronic HBV infection is 8.4%, at ≤25 age group but almost doubles within two decades (age group 25.01–35 years: 15% and age group 35.01–45 years: 16.5%). Low detection rate of anti HBc IgG antibodies (22.9%) in the ≤25 age group is also an atypical finding, although the prevalence of chronic HBV infection is over 8%. This finding allows us to consider the possibility that some patients in this age group have been infected in recent years and not in the preschool age. Anti HBc IgG positivity increases gradually in individual age groups, and reached 66.7% in patients over 45 years of age. In Asian and African areas with a high prevalence of chronic hepatitis B, only 10–20% of patients with chronic HBV infection become infected in adulthood. In the Roma settlements in East Slovakia, the prevalence of HBsAg positivity doubled and anti HBc IgG positivity tripled within two decades. The facts listed above allow us to speculate whether the horizontal transmission of HBV infection in adulthood might be the dominant way of spreading HBV infection. It is possible, that horizontal way of transmission of HBV infection in childhood has been reduced mainly because of not using disposable needles when vaccinating neonates and children in the past, what could also lead to spreading of HBV infection in that time. However, non-Roma children were vaccinated the same way; the prevalence of chronic HBV infection in the major population is several times lower than in Roma population [6]. Another reason that questions horizontal transmission of HBV infection in adulthood as the dominant way is the fact that although a significant proportion of Roma people had been having a risky behavior for HBV transmission, no significant difference was found in multivariate analysis between Roma people with chronic or past HBV infection compared to Roma people who have not been exposed to HBV infection. Only repeated testing of hepatitis B markers in the same Roma population in the future can give us a definitive answer to the role of horizontal transmission of HBV infection in adulthood.

In addition to risk factors that are directly related to the transmission of HBV infection, other factors may also play an important role. Roma people from segregated settlements live in very bad socio-economic conditions and have a less healthy lifestyle than the majority population [23,24]. Roma people have barriers to access the health care, are less likely to attend general practitioners and less frequently attend check-ups compared to the majority population [25,26]. This contributes to the fact that chronic HBV infection in the vast majority of Roma people is neither diagnosed nor treated, and these patients remain a potential source of infection for other Roma settlement residents.

There are currently two ways to prevent the spread of HBV infection in the population: i) nationwide HBV vaccination; ii) nationwide screening and treatment of chronically infected patients. A major preventive measure against the spread of HBV infection is nationwide vaccination. Mandatory vaccination of newborns in Slovakia was included in legislation in 1998 [27]. However, in some Roma children, vaccination is not completed due to the non-compliance of parents. Today, there are two treatment options for chronic hepatitis B—treatment with nucleot(s)ides analogues or PEG IFN alpha 2a [28]. Roma people compliance in the treatment with PEG IFN alpha 2a is suboptimal, which is associated with achieving a poorer virologic response [29]. At present, a long-term treatment with nucleot(s)ides analogues (entecavir, tenofovir, tenofovir alafenamide), which leads to suppression of HBV infection in near to all infected patients is preferred [28]. The global WHO strategy developed in 2016 aims at 90% reduction in the incidence of chronic hepatitis B and a 65% reduction in mortality from chronic hepatitis B [30]. The only way to try to meet this ambitious goal is to perform a screening of hepatitis B in the Roma population and to treat indicated patients with chronic hepatitis B. Patients who have not been in contact with HBV infection (antiHBc IgG-negative patients) should be vaccinated against HBV.

The biggest limitation of our study is its cross-sectional design. Another limitation is the small number of patients overall and in each age group, especially the small number of patients with chronic HBV infection. Another limitation of the study is that we did not observe the prevalence of chronic hepatitis B virus infection in the pediatric population; it should be noted here that most Roma children were vaccinated in the first year of life at the time of study. A small limitation of the study is, that we considered all HBsAg-positive patients to be chronically infected, although a minimal part of them could have had an acute hepatitis B infection. However, no patient had clinical symptoms of acute hepatitis or elevated hepatic test typical for the disease. On the contrary, the greatest benefit of the study was that we mapped the prevalence of chronic HBV infection in each age group and found that horizontal transmission of HBV infection in segregated Roma population plays a fundamental role in adulthood.

## 5. Conclusions

The prevalence of chronic HBV infection in Roma population living in settlements in East Slovakia is high and increases with the age of patients. Horizontal transmission of HBV infection in adulthood may play an important role in the HBV infection spreading. Population screening and treatment of indicated patients as well as vaccination of patients who have not been in contact with HBV infection may reduce the prevalence of HBV infection in this high-risk population in the future.

## Figures and Tables

**Table 1:** Prevalence of hepatitis B worldwide (adjusted according to [2,15]).

	High Prevalence of Hepatitis B	Moderate Prevalence of Hepatitis B	Low Prevalence of Hepatitis B
Prevalence of chronic infection	8%–15%	2%–7%	0.1%–1%
Prevalence of past infection	40%–90%	16%–55%	4%–15%
Perinatal infection	Frequent (more than 20%) *	Infrequent (10%–60%) *	Rare (less than 10%) *
Early young age at the time of infection	Very frequent (more than 60%) ^H^	Frequent (10%–60%) ^H^	Rare (less than 10%) ^H^
Adolescent and adult age at the time of infection	Infrequent (10%–20%)	Frequent (20%–50%)	Very frequent (70%–90%)
Geographic distribution	Southeast Asia, China, Pacific islands, Sub-Saharan Africa, Alaska (Eskimo)	Mediterranean sea, Eastern Europe, Central Asia, Japan, South and Latin America, Middle East	USA, Canada, Western Europe, Australia, New Zealand

* estimated percentage of total infection among children aged under 1 years old; ^H^ estimated percentage of total infection among children aged between 1 and 5 years old.

**Table 2:** Description of the whole cohort.

	Absolute(Relative) Count or Median ± IQR (Where Noted)	95% CI
Imprisonment	46 (10.3%)	7.8–12.5
IVDU	2 (0.5%)	0.08–1.8
Sex for money	13 (3%)	1.7–5
Condom use always/almost always	41 (9.3%)	0.7–12.4
>4 sexual partners	52 (11.9)	9.2–15.3
Tattoo total	173 (39.1%)	34.7–43.8
Tattoo privately	164 (37.1%)	32.7–41.7
Blood transfusion	71 (16.7%)	13.4–20.5
Hepatitis B vaccination	15 (3.5%)	2.1–4.5
HBsAg positivity	55 (12.4%)	9.7–15.8
Anti HBc IgG positivity	233 (52.8%)	48.2–57.4
Persons in living unit (median ± IQR)	7 ± 4	

IQR—interquartile range. IVDU—Intravenous drug user.

**Table 3:** Characterisation of individual age groups.

Age Group	≤25 Years	25.01–35 Years	35.01–45 Years	>45 Years	Sig
Male sex	36 (42.4%)	45 (34.4%)	56 (34.1%)	22 (32.8%)	0.541
Age (Mean ± Std. deviation)	21.8 ± 1.9	29.8 ± 3.1	39.6 ± 2.2	48.3 (2.2%)	<0.0001
Imprisonment	6 (7.1%)	10 (7.7%)	20 (12.3%)	10 (14.9%)	0.242
IVDU	1 (1.2%)	0	1 (0.6%)	0	0.994
Sex for money	1 (1.2%)	7 (5.4%)	3 (1.9%)	2 (3.0%)	0.432
Condom use always/almost always	11 (13.4%)	15 (11.8%)	12 (7.4%)	3 (4.5%)	0.165
>4 sexual partners	14 (17.1%)	18 (14.3%)	10 (6.3%)	10 (14.9%)	0.04
Tattoo total	30 (35.7%)	35 (26.9%)	69 (43.1%)	38 (56.7%)	0.0003
Tattoo privately	29 (34.5%)	31 (23.8%)	66 (41.3%)	37 (55.2%)	0.0001
Blood transfusion	6 (7.7%)	23 (18.5%)	30 (18.8%)	12 (20.0%)	0.123
Hepatitis B vaccination	2 (2.4%)	5 (3.8%)	5 (3.0%)	3 (4.5%)	0.657
Persons in living unit (median ± IQR%)	6 ± 5	6 ± 4	7 ± 4	6 ± 4	0.286

IQR—interquartile range. IVDU—Intravenous drug user.

**Table 4:** A comparison of imprisonment, drug use, blood transfusion, sexual behavior and tattoo procedure between HBsAg-positive and -negative Roma people.

	*n*	HBsAg Positive		HBsAg Negative		*p*	Multivariate Analysis OR and Significance
Male sex	442	28	50.9%	127	32.8%	0.01	2.083 (95% CI 1.176−3.690); *p* = 0.012 **
Imprisonment	435	7	12.7%	37	9.7%	0.475	
Drugs total	439	1	1.9%	9	2.3%	1	
Drugs iv	432	0	0.0%	2	0.5%	1	
>4 sexual partners	427	8	14.5%	43	11.6%	0.507	
Sex for money	429	2	3.6%	11	2.9%	0.677	
Tattoo total	432	23	44.2%	147	38.7%	0.453	
Tattoo private	432	20	38.5%	141	37.1%	0.879	
Tattoo parlor	432	3	5.8%	6	16.8%	0.082	
Blood transfusion	418	8	14.8%	61	16.8%	0.845	
Age(years) *	442	35.24 (12.17)		35.5 (15.5)		0.864	

* Median (IQR)]; IQR—interquartile range; significance tested by Fisher exact test or Mann–Whitney U test where appropriate. OR—odds ratio; multivariate ORs calculated by logistic regression adjusted for age **. *n*—number of evaluated patients.

**Table 5:** A comparison of imprisonment, drug use, blood transfusion, sexual behavior and tattoo procedure between anti HBc IgG-positive and anti HBc IgG-negative Roma people.

	*n*	Anti HBcIgG Positive		Anti HBcIgG Negative		*p*	Multivariate ORs and Significance
Male sex	441	91	39.10%	64	60.8%	0.069	
Imprisonment	434	31	13.50%	13	6.4%	0.014	1.600; (95% CI 0.702–3.646); *p* = 0.263 **
Drugs total	438	3	1.30%	6	2.9%	0.317	
Drugs iv	431	1	0.40%	1	0.5%	1	
>4 sexual partners	426	23	10.20%	28	14.0%	0.225	
Sex for money	428	8	3.50%	5	2.5%	0.533	
Tattoo total	431	100	44.20%	69	33.7%	0.025	1.070; (95% CI 0.663–1.727); *p* = 0.781 **
Tattoo private	431	95	42.00%	65	31.7%	0.027	1.163; (95% CI 0.704–1.833); *p* = 0.601 **
Tattoo parlor	431	5	2.20%	4	2.0%	1	
Blood transfusion	417	35	15.80%	33	16.8%	0.783	
Age(years) *	436	37.7 (11.98%)		30.75 (16.12)		<0.0001	OR for 1-year increment was 1.080; (95% CI 1.054–1.107); *p* < 0.0001 ***

* Median (IQR); IQR—interquartile range; significance tested by Fisher exact test or Mann–Whitney U test where appropriate. ORs—odds ratios; Multivariate ORs calculated by logistic regression adjusted for age and sex ** or for sex ***. *n*—number of evaluated patients.

## Abbreviations

Anti HBc	Antibodies to Hepatitis B core antigen
Anti HBe	Antibodies to Hepatitis B e antigen
Anti HBs	Antibodies to Hepatitis B surface antigen
HBV	Hepatitis B virus
HBeAg	Hepatitis B e antigen
HBsAg	Hepatitis B surface antigen
HCC	Hepatocellular cancer
IgG	Immunoglobulin G
IVDU	Intravenous drug user
PEG	IFN Pegylated interferon

## Appendix A

HepaMeta Team members: Peter Jarcuska, Andrea Madarasova Geckova, Mária Marekova, Daniel Pella, Leonard Siegfried, Pavol Jarcuska, Lydia Pastvova, Ján Fedacko, Jana Kollarova, Peter Kolarcik, Daniela Bobakova, Zuzana Dankulincova Veselska, Ingrid Babinska, Sylvia Drazilova, Jaroslav Rosenberger, Ivan Schreter, Pavol Kristian, Eduard Veseliny, Martin Janicko, Ladislav Virag, Anna Birkova, Marta Kmetova, Monika Halanová, Darina Petrasova, Katarína Carikova, Viera Lovayova, Lucia Merkovska, Lucia Jedlickova, Ivana Valkova.

## References

[1] Fattovich G. (2003). Natural history of hepatitis B. J. Hepatol..

[2] Alter M.J. (2003). Epidemiology of hepatitis b in europe and worldwide. J. Hepatol..

[3] Nelson N.P., Easterbrook P.J., McMahon B.J. (2016). Epidemiology of hepatitis B virus infection and impact of vaccination on disease. Clin. Liver Dis..

[4] Alter M.J., Margolis H.S. (1990). The emergence of hepatitis B as a sexually transmitted disease. Med. Clin. North Am..

[5] Lok A.S., McMahon B.J. (2007). Chronic hepatitis B. Hepatology.

[6] Drazilova S., Janicko M., Kristian P., Schreter I., Halanova M., Urbancikova I., Madarasova-Geckova A., Marekova M., Pella D., Jarcuska P. (2018). Prevalence and risk factors for hepatitis B virus infection in Roma and non-Roma people in Slovakia. Int. J. Environ. Res. Public Health.

[7] Stevens C.E., Neurath R.A., Beasley R.P., Szmuness W. (1979). Hbeag and anti-HBe detection by radioimmunoassay: Correlation with vertical transmission of hepatitis B virus in Taiwan. J. Med. Virol..

[8] Xu Z.Y., Liu C.B., Francis D.P., Purcell R.H., Gun Z.L., Duan S.C., Chen R.J., Margolis H.S., Huang C.H., Maynard J.E. (1985). Prevention of perinatal acquisition of hepatitis B virus carriage using vaccine: Preliminary report of a randomized, double-blind placebo-controlled and comparative trial. Pediatrics.

[9] Polaris Observatory C. (2018). Global prevalence, treatment, and prevention of hepatitis b virus infection in 2016: A modelling study. Lancet Gastroenterol. Hepatol..

[10] Tanaka J., Akita T., Ko K., Miura Y., Satake M. (2019). Epidemiological Research Group on Viral Hepatitis and its Long -term Course, Ministry of Health, Labour and Welfare of Japan. Countermeasures against viral hepatitis B and C in Japan: An epidemiological point of view. Hepatol. Res..

[11] Hou J., Liu Z., Gu F. (2005). Epidemiology and prevention of hepatitis B virus infection. Int. J. Med. Sci..

[12] Shepard C.W., Simard E.P., Finelli L., Fiore A.E., Bell B.P. (2006). Hepatitis B virus infection: Epidemiology and vaccination. Epidemiol. Rev..

[13] Andre F. (2000). Hepatitis B epidemiology in Asia, the Middle East and Africa. Vaccine.

[14] Tanaka J. (2000). Hepatitis B epidemiology in Latin America. Vaccine.

[15] Maynard J.E. (1990). Hepatitis B: Global importance and need for control. Vaccine.

[16] Gecková A.M., Jarcuska P., Mareková M., Pella D., Siegfried L., Jarcuska P., Halánová M. (2014). Hepameta—Prevalence of hepatitis B/C and metabolic syndrome in population living in separated and segregated Roma settlements: A methodology for a cross-sectional population-based study using community-based approach. Cent. Eur. J. Public Health.

[17] Do S. (2001). The natural history of hepatitis B in Asian Americans. Asian Am. Pac. Isl. J. Health.

[18] Minuk G.Y., Bohme C.E., Bowen T.J., Hoar D.I., Cassol S., Gill M.J., Clarke H.C. (1987). Efficacy of commercial condoms in the prevention of hepatitis B virus infection. Gastroenterology.

[19] Bernabe-Ortiz A., Carcamo C.P., Scott J.D., Hughes J.P., Garcia P.J., Holmes K.K. (2011). HBV infection in relation to consistent condom use: A population-based study in Peru. PLoS ONE.

[20] Kirwan P., Evans B., Sentinel Surveillance of Hepatitis Testing Study G., Brant L. (2011). Hepatitis C and B testing in English prisons is low but increasing. J. Public Health.

[21] Jafari S., Buxton J.A., Afshar K., Copes R., Baharlou S. (2012). Tattooing and risk of hepatitis B: A systematic review and meta-analysis. Can. J. Public Health.

[22] Zampino R., Boemio A., Sagnelli C., Alessio L., Adinolfi L.E., Sagnelli E., Coppola N. (2015). Hepatitis B virus burden in developing countries. World J. Gastroenterol..

[23] Geckova A.M., Babinska I., Bobakova D., Veselska Z.D., Bosakova L., Kolarcik P., Jarcuska P., Pella D., Halanova M., HepaMeta Team (2014). Socioeconomic characteristics of the population living in Roma settlements and their association with health and health-related behaviour. Cent. Eur. J. Public Health.

[24] Babinska I., Geckova A.M., Jarcuska P., Pella D., Marekova M., Stefkova G., Veselska Z.D., HepaMeta, Team (2014). Does the population living in Roma settlements differ in physical activity, smoking and alcohol consumption from the majority population in Slovakia?. Cent. Eur. J. Public Health.

[25] Jarcuska P., Bobakova D., Uhrin J., Bobak L., Babinska I., Kolarcik P., Veselska Z., Madarasova Geckova A., HEPA-META Team (2013). Are barriers in accessing health services in the Roma population associated with worse health status among roma?. Int. J. Public Health.

[26] Dolak F., Sedova L., Novakova D., Olisarova V. (2016). Approach to prevention of obesity of Roma population in the region of south bohemia with focus on selected eating behaviors. Neuro Endocrinol. Lett..

[27] Veseliny E., Janicko M., Drazilova S., Siegfried L., Pastvova L., Schreter I., Kristian P., Viag L., Jarcuska P., Valkova I. (2014). High hepatitis B and low hepatitis C prevalence in Roma population in Eastern Slovakia. Cent. Eur. J. Public Health.

[28] European Association for the Study of the Liver (2017). EASL 2017 clinical practice guidelines on the management of hepatitis B virus infection. J. Hepatol..

[29] Drazilova S., Janicko M., Kristian P., Schreter I., Kucinsky B., Kozlej M., Hockickova I., Jarcuska P. (2016). Lower viral response to pegylated interferon alpha 2a treatment of chronic hepatitis b in Roma people in Eastern Slovakia. Gastroenterol. Res. Pract..

[30] Spearman C.W., Afihene M., Ally R., Apica B., Awuku Y., Cunha L., Dusheiko G., Gogela N., Kassianides C., Kew M. (2017). Hepatitis B in Sub-saharan Africa: Strategies to achieve the 2030 elimination targets. Lancet Gastroenterol. Hepatol..

